# Identification and antimicrobial resistance profiling of *Pseudomonas aeruginosa* using multi-excitation Raman spectroscopy and computational analytics

**DOI:** 10.1038/s44259-025-00141-z

**Published:** 2025-08-25

**Authors:** Callum Highmore, Niall Hanrahan, Yoshiki Cook, Ysanne Pritchard, Adam Lister, Kirsty Cooper, George Devitt, Alasdair P. S. Munro, Saul N. Faust, Sumeet Mahajan, Jeremy S. Webb

**Affiliations:** 1 School of Biological Sciences, Faculty of Environmental and Life Sciences, University of Southampton, SO17 1BJ Southampton, UK; 2 National Biofilms Innovation Centre (NBIC) and Institute for Life Sciences, University of Southampton, SO17 1BJ Southampton, UK; 3 NIHR Southampton Clinical Research Facility and Biomedical Research Centre, University Hospital Southampton NHS Foundation Trust, Southampton, SO16 6YD UK; 4 Institute for Life Sciences, University of Southampton, University of Southampton, SO17 1BJ Southampton, UK; 5 School of Chemistry and Chemical Engineering, Faculty of Engineering and Physical Sciences, University of Southampton, SO17 1BJ Southampton, UK; 6 School of Mathematical Sciences, Faculty of Social Sciences, University of Southampton, SO17 1BJ Southampton, UK; 7 Faculty of Medicine and Institute for Life Sciences, University of Southampton, Southampton, SO17 1BJ UK

**Keywords:** Antimicrobial resistance, Infectious-disease diagnostics, Optics and photonics

## Abstract

Antimicrobial resistance (AMR) poses a global healthcare challenge, where overprescription of antibiotics contributes to its prevalence. We have developed a rapid multi-excitation Raman spectroscopy methodology (MX-Raman) that outperforms conventional Raman spectroscopy and enhances specificity. A support vector machine (SVM) model was used to identify 20 clinical isolates of *Pseudomonas aeruginosa* with an accuracy of 93% using MX-Raman. Antibiotic sensitivity profiles for tobramycin, ceftazidime, ciprofloxacin, and imipenem were generated for the bacterial strains and compared with their Raman spectral signatures using MX-Raman. The 20 clinical strains were distinguished according to AMR profiles. Nine models were assessed for AMR classification performance, and SVM performed best, classifying AMR profiles of each strain with 91–96% accuracy. These data provide the basis for a new rapid clinical diagnostic platform that could screen for bacterial infection and recommend effective antibiotic treatment ahead of confirmation by conventional techniques, improving clinical outcomes and reducing the spread of AMR.

## Introduction

Antimicrobial resistance (AMR) is among the most significant global health threats to humanity, where an estimated 4.95 million deaths were associated with AMR in 2019, and by 2050 AMR is expected to cause 10 million deaths annually^1,2^. Slow, inefficient antimicrobial sensitivity tests exacerbate this problem. Antibiotic sensitivity tests (AST) continue to rely on culture techniques that require 48 h to determine an AMR profile, leading to the use of presumptive antibiotic therapies. It has been estimated that at least 20% of antibiotic prescriptions in primary care are inappropriate^3^, further driving the development of AMR. One study determined that 32.4% of children visiting one UK hospital were prescribed antibiotics, however, only 7.1% were diagnosed with bacterial infection. The costs associated with unnecessary antibiotic treatments were calculated at an additional £1352.10, primarily in inpatient-care costs per child while awaiting AST results^4^.

Multiple existing and emerging technologies for pathogen identification and AMR profiling aim to reduce time to diagnosis and improve treatment efficacy. Examples include nucleic acid amplification, genomic and metagenomic sequencing approaches, immunodiagnostic arrays and mass spectrometry-based methods^5–9^. Microfluidic-based diagnostics are also increasingly considered as future infection diagnostic technologies, as they are often cost-effective and provide single-cell analysis^10–12^. Despite an increasing number of molecular and point-of-care diagnostic technologies, the identification of pathogens and AMR within large and complex microbial communities present at an infection site remains challenging.

Raman spectroscopy is currently being investigated as an alternative diagnostic technology that includes the potential for real-time, in situ identification of pathogens within complex samples such as chronic wounds, persistent or recurrent respiratory or device-related infections^13–15^. By measuring changes in the vibrational modes of bacterial samples according to taxonomic and phenotypic differences^13,14,16,17^, Raman spectroscopy offers rapidity and requires minimal sample preparation. While commonly cited drawbacks to the technology include limits to sensitivity, several approaches have been studied to overcome this. Surface-enhanced Raman spectroscopy (SERS) improves the sensitivity of the Raman signal but increases sample preparation. SERS has successfully distinguished 14 strains of *Arthrobacter* strains with 97% accuracy^18^. Label-based SERS further improves sensitivity by binding specific aptamers to the bacterial cell surface. Li et al.^19^ combined gold nanorod-based SERS tags with antibody-modified magnetic nanoparticles for the detection of pathogens in food samples. This process facilitated the identification of prominent bacterial foodborne pathogens from food samples at low cell concentrations, e.g. 5 CFU/ml for *E. coli* O157:H7. SERS methodologies have been applied to AST^20,21^; 89 *E. coli* strains were separated according to their sensitivity to carbapenems via subtle differences in the Raman spectral signatures of the bacteria, amplified by SERS processing^20^.

Previously, we have developed a Raman-based methodology, multi-excitation Raman spectroscopy (MX-Raman), which utilises multiple laser wavelengths to improve the specificity of Raman spectroscopy and demonstrated that this was possible within a complex artificial sputum medium^13^. We have also demonstrated the utility of Raman spectroscopy in AMR detection when coupled with genome sequencing^22^.

The aim of this study was to demonstrate the utility of MX-Raman, combined with robust and optimised computational analyses, to accurately identify and profile AMR among a panel of 20 clinical *Pseudomonas aeruginosa* respiratory infection isolates. AMR profiles across a range of antibiotic classes were compared against MX-Raman spectral signatures using nine machine learning models to identify the best method for AMR classification. Our study demonstrated accurate (93%) classification of clinical isolates and their associated AMR profile suggesting the potential future utility of this technique for novel in situ diagnostic approaches.

## Results

### A Raman spectral library was collected for 20P. aeruginosa clinical isolate strains

**Fig. 1:**
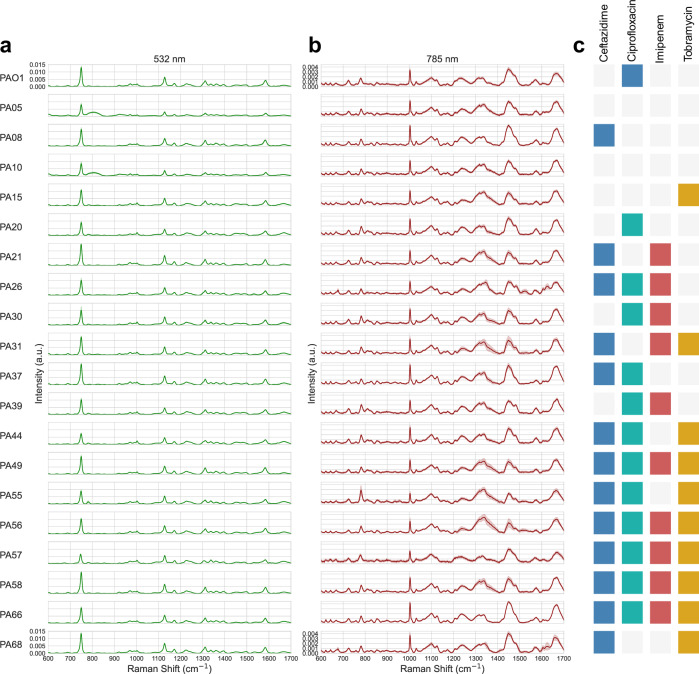
Raman spectral library of 20 *Pseudomonas aeruginosa* clinical isolates and their associated sensitivities to four antibiotics. A Raman spectral library of 20 *Pseudomonas aeruginosa* strains was acquired using both (**a**) 532 nm and (**b**) 785 nm laser excitations. **c** Minimum inhibitory concentration testing was conducted to assign a binary sensitivity label (sensitive or resistant) to each strain following exposure to four antibiotics: ceftazidime, ciprofloxacin, imipenem, and tobramycin. The mean spectrum and standard deviation of each strain and excitation wavelength following spectral pre-processing are displayed alongside their strain and sensitivity labels. The resistance of each strain to each antibiotic: ciprofloxacin, ceftazidime, imipenem, and tobramycin, are indicated by the colours: blue, teal, red, and yellow, respectively.

Raman spectra were collected for each *P. aeruginosa* strain, using lasers at both 532 nm (Fig. 1a) and 785 nm (Fig. 1b) excitations. The spectral differences between strains are apparent and are increased by the multi-excitation approach—where we combine the data obtained with each of the excitation wavelengths of 532 nm and 785 nm. While a 785 nm excitation generates a consistent peak at 1003 cm^−1^ (indicating phenylalanine), excitation at 532 nm at the same wavenumber, results in variability between the 20 strains (Fig. 1). The Raman shift at 1128 cm^−1^ (attributed to C–C and C = C stretching modes of carotenoids and polyenes in bacterial biofilms) is present via excitation at 532 nm with varying intensity for each of the *P. aeruginosa* strains. Visible variation between the strains’ spectra is also present at 1312 and 1337 cm^−1^, indicating variation in nucleic acid content^23,24^. Variation is similarly visible in spectra acquired using 785 nm excitation, such as the peak at 782 cm^−1^ indicating differences in nucleic acid content across strains^24,25^. Peaks at 1521 cm^−1^ and 1624 cm^−1^ using 785 nm excitation are particularly prominent for PA26, and to a lesser extent in PA68 and PA57. These are also the strains with the most intra-strain variability in their spectra, and produce the pigment pyocyanin^26^. Pyocyanin could be responsible for these peaks due to its C = C bonds in its tricyclic rings, and the fluorescence generated by these compounds could cause the measured intra-strain variability^27^ (Fig. 1b). Peaks present in PA05 and PA10 at 800 cm^−1^ are consistent with glass (Fig. 1a)^28^.

To further investigate the variation between the *P. aeruginosa* strains, PCA was conducted on the Raman spectral datasets acquired using the 532 nm and 785 nm excitations on their own (single-excitation), and in combination via the MX-Raman dataset (i.e. the data acquired from excitation at both 532 nm and 785 nm) (Supplementary Fig. 1). Using the first three principal components (PCs), a cluster analysis was performed to observe the separation between spectra of the same strain. Overall, each strains’ spectra were found to cluster together, with some smaller subclusters present within each strain correlating to biological repeats of the bacterial strains. The presence of peaks attributed to pyocyanin in PA26 and PA68, and to glass in PA05 and PA10, is reflected in the PCA (Supplementary Fig. 2).

### Model performance was analysed for optimal clinical isolate strain classification

**Fig. 2:**
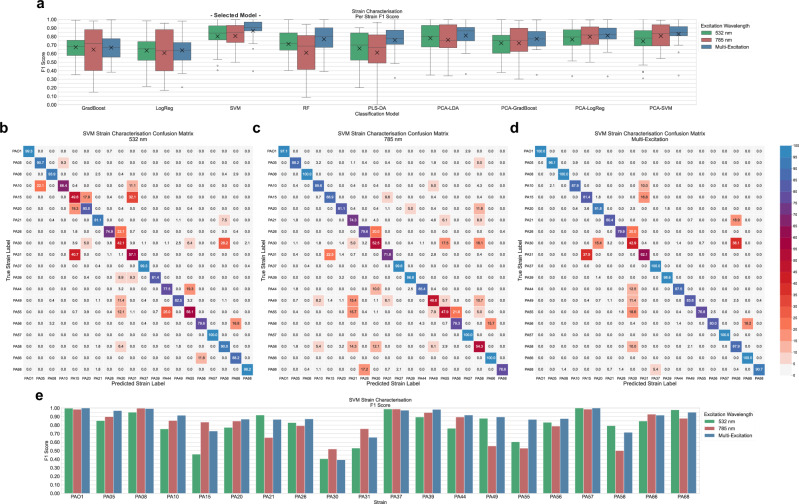
Classification performances for strain identification of 20 *Pseudomonas aeruginosa* clinical isolates using single-excitation and multi-excitation raman spectroscopy. **a** Nine machine learning classifiers (GradBoost, LogReg, SVM, RF, PLS-DA, PCA-LDA, PCA-GradBoost, PCA- LogReg, PCA-SVM) were applied to each of the three Raman spectral datasets (532 nm, 785 nm, and multi-excitation) for strain identification of 20 *Pseudomonas aeruginosa* clinical isolates. Across all classifiers, the multi-excitation approach was found to outperform both single-excitation approaches with respect to the macro-averaged F1 score (indicated by an ‘X’). The highest performing of these, SVM, achieved a macro-averaged F1 score of 0.80, 0.81, and 0.87 for the 532 nm, 785 nm, and multi-excitation Raman excitations, respectively, and was selected among the nine classifiers for further investigation. The confusion matrices for the (**b**) 532 nm, (**c**) 785 nm, and (**d**) multi-excitation Raman datasets using the SVM algorithm were used to compare strain label assignments across the three approaches. Strains uniquely mislabelled by the single-excitation approaches were found to be corrected using the combined multi-excitation dataset. **e** The per class F1 score for each dataset was also compared, to evaluate performance and stability for both strains and excitation wavelengths. Overall, the multi-excitation approach was found to outperform or match the highest performing single-excitation approach with respect to per strain accuracy and F1 score for 11 out of the 20 investigated strains. In all other cases, the multi-excitation approach achieved the second-best performance, out of the three datasets, save for PA30 which classified poorly across all approaches.

To determine the highest performing model in discriminating between the 20 clinical isolates, nine classifiers were trained on both single-excitation (532 and 785 nm) and the combined multi-excitation spectral datasets (Fig. 2). For each investigated classifier, the MX-Raman dataset was found to outperform both single-excitation Raman approaches with respect to the adjusted F1 score. The highest performing of these was the SVM classifier which achieved a macro mean F1 of 0.87 and standard deviation of 0.15, for an adjusted F1 score of 0.72 for the MX-Raman dataset (Fig. 2a, Table 1).

**Table 1:**
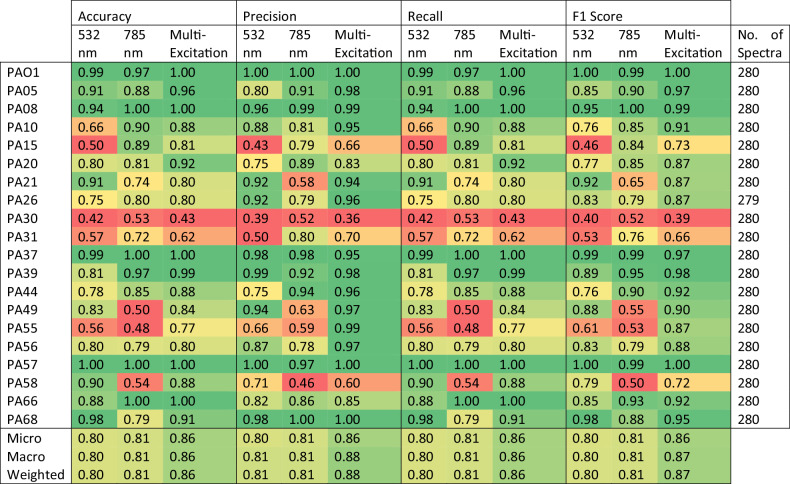
Strain Identification SVM Classification Performances using Single-Excitation and Multi-Excitation Raman Spectroscopy

SVM was selected as the highest performing classification model (with respect to the adjusted F1 score) for strain identification using single- (532 nm, and 785 nm) and multi-excitation Raman spectroscopy. The per strain classification performances (Accuracy, Precision, Recall, and F1 score) and their averages (Micro, Macro, and Weighted) across the three approaches were compared for granular assessment. The number of spectra used in the Leave-One-Biological-Replicate-Out cross-validation for each strain (No. of Spectra) are also reported for cases of class imbalance.

As the best algorithm for all spectral datasets, the SVM models were further examined with respect to the other recorded metrics (accuracy, precision, and recall) as well as the precise label assignments made in classifying the *P. aeruginosa* strains. The MX-Raman dataset performed consistently better than single-excitation datasets using SVM, with respect to model accuracy (0.86), precision (0.88), recall (0.86), and F1 score (0.87) (Table 1). The MX-Raman approach also gave the highest overall performance across individual strains, with F1 scores either greater than or equal to 55% of the single-wavelength equivalents (Fig. 2e). The F1 macro score was used as the key determinant of model viability due to it balancing model precision and recall. For strains where a single-excitation score exceeded the MX-Raman score, the difference is marginal. The exception is PA30, where the MX-Raman F1 score is 0.39, compared to 0.4 and 0.52 for analysis with the single-excitation of 532 nm and 785 nm, respectively (Table 1). The SVM confusion matrices show that there are significant and distinct misclassifications for PA30 for both single-excitation data analyses, which are not compensated for in the MX-Raman analysis and even leads to an increased misclassification with PA58 spectra (Fig. 2).

The confusion matrices exhibit variation between the classification accuracy of strains; the MX-Raman matrix ranges in classification accuracy from 42.9% (PA30) to 100%, with 87.9% as the median. There is a high degree of misclassification of PA31 in the single-excitation analyses at 532 nm and 785 nm, where it has been misclassified as PA15 at 40.7% and 22.5%, respectively. The misclassification remains at 37.9% in the MX-Raman analysis (Fig. 2d). PA55 has significant misclassifications in both single-excitation analyses with accuracies of 56.1% and 47.9%, however, these are resolved in the MX-Raman analysis with a classification accuracy of 76.8%.

Bacterial identification at the strain level is not typically conducted in the clinic; however, we considered this an important aspect of our study as it demonstrates that there are perceivable differences in Raman spectra between the clinical isolates ahead of their categorisation according to their AMR profiles. Strain level categorisation will also be more important as this methodology is applied to other bacterial species in future work, where the definition of strains within pathogen species groups will ensure the establishment of appropriate classification boundaries in computational models.

To compare the discriminative capabilities of MX-Raman to traditional single-excitation approaches, two single-excitation spectral datasets (acquired using a 532 and 785 nm laser excitation) and one multi-excitation dataset (combining the 532 and 785 nm spectra) were used to discriminate between 20 clinical isolates of *P. aeruginosa*. Nine classifiers were trained on each of the three spectral datasets and, amongst these, the classifier with the greatest adjusted F1 score across all datasets was selected to balance both high average performance and low variability in scores across strains. By this procedure, the multi-excitation approach was found to outperform (or equal in the case of the LogReg classifier) all equivalent single-excitation approaches across all investigated classifiers—save for the GradBoost algorithm where the multi-excitation dataset matched the 532 nm dataset in terms of the macro F1 score (both 0.67), but was marginally beaten by the single-excitation approach due to a 0.01 difference in standard deviation (Supplementary Table 1). The highest performing of all trained classifiers was the SVM algorithm, which achieved an adjusted F1 score of 0.63, 0.64, and 0.72 when using the 532 nm, 785 nm, and multi-excitation Raman dataset, respectively (Supplementary Table 1). As the best algorithm of the nine classifiers, the three SVM models were further investigated to compare all other recorded metrics (i.e. accuracy, precision, and recall), per strain performances, and the precise class label assignments selected by each of the models. SVM models were further investigated to compare all other recorded metrics (i.e. accuracy, precision, and recall), per strain performances, and the precise class label assignments selected by each of the models. Models were further investigated to compare all other recorded metrics (i.e. accuracy, precision and recall), per strain performances, and the precise class label assignments selected by each of the models.

Throughout all macro-averaged scores, the MX-Raman SVM model consistently exceeded those achieved by the 532 nm and 785 nm spectral datasets alone. The combined approach was found to not only increase the macro-averaged F1 score and reduce its variability across strains (achieving a score of 0.80 ± 0.17, 0.81 ± 0.17, and 0.87 ± 0.15, for the 532 nm, 785 nm, and multi-excitation datasets, respectively), but also increase the overall accuracy (0.80, 0.81, and 0.86), precision (0.81, 0.81, and 0.88), and recall (0.80, 0.81, and 0.86) (Supplementary Table 1, Table 1, Fig. 2a). Beyond averaged performance metrics, the multi-excitation dataset was also found to have an improved F1 score, over the 532 nm and 785 nm datasets, for 11 out of the 20 strains (Table 1 Fig. 2). In all other cases, where a single-excitation approach achieved the best F1 score for a particular strain, the multi-modal technique accomplished an often-marginal reduction in performance in comparison to the best dataset (Fig. 2e). The exception to this was the strain PA30, where the multi-excitation Raman approach scored the lowest F1 score of the three methods, achieving a value of 0.39 compared to the values 0.40, and 0.52 scored by the 532 nm, and 785 nm dataset, respectively (Table 1, Fig. 2e). PA30 was also identified as the one of the lowest performing of the 20 strains for all spectral datasets.

To further evaluate the differences between models, the exact label assignments made by the SVM algorithm for each approach were investigated using the associated confusion matrices (Fig. 2b–d). As the confusion matrix provides a detailed account of how each spectrum of a given strain was labelled by the SVM classifier, examining these heatmaps provides a graphical method for identifying common misclassifications made within and across each model. Figure 2b–d shows the confusion matrix obtained using the SVM classifier trained on the 532 nm, 785 nm, and MX-Raman spectral datasets, respectively. Each row of the confusion matrix describes the distribution of predicted labels across a given strain. The confusion matrix of an ideal model—able to discriminate perfectly between all strains without misclassification—will display values of 100.0% on every diagonal entry and zeros elsewhere. As an example, the first row of each confusion matrix shows the proportion of spectra truly labelled as PAO1 that were labelled as each of the 20 strains (given as a percentage) by the SVM algorithm. In the case of the 532 nm model, 99.3% of all PAO1 spectra were labelled correctly as PAO1, whilst the remaining 0.7% of spectra were incorrectly predicted to be the strain PA26 (Fig. 2b). In contrast, the multi-excitation Raman approach can be seen to have categorised all 100% of the PAO1 spectra correctly (Fig. 2d).

The confusion matrix is particularly useful as it emphasises common misclassifications made by each model. Here, we defined a common misclassification to be any event for which over 5% of the spectra of a given strain class were predicted to be anything other than their true strain (indicated by a coloured off-diagonal element). Using this description, the 532 nm, 785 nm, and MX-Raman models were each found to have made a total of 21, 28, and 13 common misclassifications, respectively (Fig. 2b–d). As the multi-excitation dataset was constructed using the single-excitation spectra, the 13 misclassifications made by the combined approach were identified to be a subset of the 21, and 28 errors made by the 532 nm, and 785 nm datasets—with the exception of one minor common misclassification unique to the MX-Raman approach in which 5.4% of the PA68 spectra were mislabelled as PA37 (Fig. 2d). Many of the common misclassifications made by the single-excitation models were found to be greatly reduced when using the multi-excitation approach. One example of this can be seen for the strain PA55, where 25.0% of all PA55 spectra were mislabelled as PA44 in the 532 nm model, and only 0.4% of PA55 spectra were mislabelled as such in the MX-Raman model (Fig. 2b, d). A similar improvement was also seen for the 785 nm model, where 21.8% of PA55 spectra were misassigned the label PA56 in the 785 nm model, and only 2.5% of PA55 spectra were predicted as PA56 when using the multi-excitation approach (Fig. 2b, d).

The largest misclassifications in the combined approach were found to involve the strains PA30 and PA31—either through mislabelling PA30, and PA31 as other strains or vice versa (Fig. 2d). The single-excitation approaches were also found to struggle most with distinguishing these strains from all others (Fig. 2b, c). As both the 532 nm and 785 nm models mislabelled a high proportion of PA31 and PA30 strains as PA15, and PA58, respectively, these errors were found to persist in the combined MX-Raman approach and identified as the highest misclassifications in the model (Fig. 2d).

Overall, MX-Raman was found to improve the discriminative capability of Raman spectroscopy for strain-level characterisation of 20 clinical isolates of *P. aeruginosa*. Models were scrutinised with an appropriate metric to balance both high average performance and low variability in characterizability across strains. The MX-Raman approach was found to improve the per strain accuracy for the majority of strains over the single-excitation counterparts, and many common misclassifications were found to be reduced when combining information from both 532 nm and 785 nm excitation spectra. Although bacterial identification at the strain level is not typically conducted in the clinic, we considered this an important aspect of our study as it demonstrates that there are perceivable differences in Raman spectra between the clinical isolates ahead of their categorisation according to their AMR profiles. Strain level categorisation will also be more important as this methodology is applied to other bacterial species in future work, where the definition of strains within pathogen species groups will ensure the establishment of appropriate classification boundaries in computational models.

### Clinical Isolates were classified according to their antibiotic sensitivity profiles using MX-Raman

MICs for each *P. aeruginosa* strain were raised for tobramycin, ceftazidime, ciprofloxacin, and imipenem (Table 2, Supplementary Table 2), representing different antibiotic classes. For tobramycin and imipenem, 50% of the strains were categorised as resistant in accordance with EUCAST guidelines. For ceftazidime and ciprofloxacin, 65%, and 25% of strains were categorised resistant, and sensitive, respectively. PA49, PA56, PA57, PA58 and PA66 were resistant to each antibiotic tested, and PA05 and PA10 were sensitive to each antibiotic tested. These similarities were not reflected in misclassifications in the SVM strain classification model (Fig. 2b–d).

**Table 2:** Minimum inhibitory concentration tests were conducted to identify the sensitivities of 20 clinical isolates of *Pseudomonas aeruginosa* to the antibiotics: ceftazidime, ciprofloxacin, imipenem, and tobramycin

	Tobramycin		Ceftazidime		Ciprofloxacin		Imipenem	
	MIC	S/R	MIC	S/R	MIC	S/R	MIC	S/R
PAO1	0.33	S	3.33	S	0.67	R	2.67	S
PA05	0.25	S	1.00	S	0.42	S	1.33	S
PA08	0.83	S	64.00	R	0.25	S	0.83	S
PA10	0.42	S	2.33	S	0.33	S	0.67	S
PA15	2.67	R	0.83	S	0.50	S	1.00	S
PA20	0.42	S	0.67	S	0.58	R	1.17	S
PA21	0.58	S	42.67	R	0.25	S	21.33	R
PA26	0.67	S	256.00	R	0.67	R	26.67	R
PA30	0.25	S	7.50	S	0.67	R	9.33	R
PA31	2.33	R	60.00	R	0.33	S	7.50	R
PA37	0.50	S	85.33	R	1.00	R	3.75	S
PA39	0.58	S	4.00	S	0.58	R	4.67	R
PA44	85.33	R	22.67	R	0.83	R	3.33	S
PA49	42.67	R	64.00	R	0.67	R	18.67	R
PA55	213.33	R	27.00	R	0.83	R	1.33	S
PA56	64.00	R	74.00	R	1.17	R	6.67	R
PA57	26.67	R	53.33	R	0.58	R	5.33	R
PA58	32.00	R	64.00	R	0.58	R	10.67	R
PA66	64.00	R	64.00	R	1.12	R	10.67	R
PA68	26.67	R	64.00	R	0.42	S	1.67	S

MICs of *P. aeruginosa* isolates (units mg/l). Presented MIC values were averaged across at least 3 repeats.

New analyses were conducted to separate the clinical isolates according to their sensitivity to each of the antibiotics tested. Both the single-excitation and MX-Raman approaches were tested against nine different machine learning classifiers. The highest performing classification algorithm was selected based on the adjusted F1 score to assess model performance for the unbalanced class size problems. In cases where the difference between the best performing models was found to be marginal, the final model was selected based on the highest macro-averaged F1 score across the three wavelengths.

**Fig. 3:**
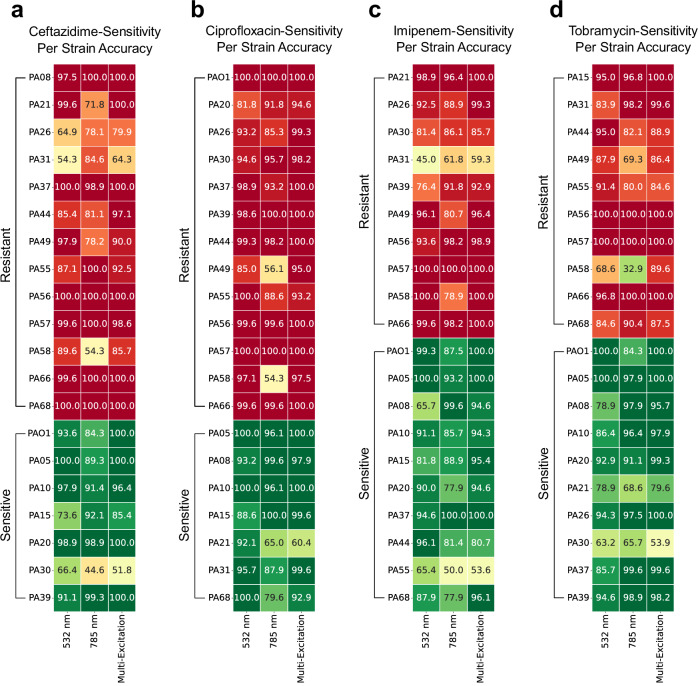
Classification accuracies for antibiotic-sensitivity profiling of 20 *Pseudomonas aeruginosa* clinical isolates using single-excitation and multi-excitation Raman spectroscopy. Nine machine learning classifiers were applied to predict the sensitivities of 20 *Pseudomonas aeruginosa* strains to four antibiotics: (**a**) ceftazidime, (**b**) ciprofloxacin, (**c**) imipenem, and (**d**) tobramycin, using both single-excitation (532 nm, and 785 nm), and multi-excitation Raman approaches. The highest performing classifier, with respect to the adjusted F1 score, was identified for each antibiotic-sensitivity classification task and selected for further investigation. Of the applied classifiers, SVM was found to be the highest performing model for all four antibiotics, with an adjusted F1 score of (**a**) 0.85, 0.81, and 0.88, (**b**) 0.94, 0.86, and 0.94, (**c**) 0.88, 0.86, and 0.92, and (**d**) 0.89, 0.87, and 0.93, for the 532 nm, 785 nm, and multi-excitation datasets, respectively. In all cases, the multi-excitation approach was found to outperform or match both single-excitation approaches. To further compare the difference in performances across the three excitation approaches, the per strain accuracies of each SVM model was evaluated. The heatmap displays the accuracy of the SVM model in correctly predicting the sensitivity of each strain using the three spectral datasets. Strains are grouped by their true sensitivity to the respective antibiotic to visualise class imbalance for each antibiotic-sensitivity characterisation task. A red–yellow–green colourmap is used to display overall classification where red represents an overall resistant classification, and green an overall sensitive classification.

The SVM classifier was identified to have the highest adjusted F1 score for determining a strains’ resistance to each of the four antibiotics, with a value range of 0.81–0.88, 0.84–0.94, 0.86–0.92, and 0.87–0.93 across the three spectral datasets for the drugs, ceftazidime, ciprofloxacin, imipenem, and tobramycin, respectively (Fig. 3, Supplementary Table 3). Across all three spectral datasets, the multi-excitation approach was found to outperform both the 532 nm, and 785 nm models with respect to this metric, achieving an adjusted F1 score of 0.88, 0.94, 0.92, and 0.93 for the ceftazidime, ciprofloxacin, imipenem, and tobramycin-sensitivity tasks, respectively (Supplementary Table 3).

For each antibiotic-sensitivity characterisation task, the per strain classification accuracy was evaluated for a more granular assessment of model performances. Figure 3 displays the classification accuracies obtained for each antibiotic-sensitivity characterisation task using both single-excitation (532 nm, and 785 nm) and multi-excitation Raman approaches via a heatmap. Each entry displays the accuracy in determining a strains sensitivity to the given antibiotic using each of the three spectral datasets. Strains are grouped by their (true) sensitivity to each antibiotic to visualise imbalanced class sizes. A green-yellow-red colour bar was applied to visualise the overall predicted sensitivity for each strain, with green representing an overall sensitive and red representing an overall resistant classification.

As seen in Fig. 3, when using the MX-Raman approach, classification of a strains’ resistance to ciprofloxacin scored particularly well, with an overall macro F1 score of 0.96 and no isolate classifying particularly poorly—seen as all strains achieving an overall sensitivity colouring matching their true sensitivity (i.e. all truly resistant strains coloured red, and all truly sensitive strains coloured green). The PA30 and PA31 isolates classify relatively poorly to ceftazidime and imipenem, and ceftazidime and tobramycin, respectively. The ceftazidime MIC of PA30 was also the closest to the 8 mg/l resistance boundary, at 7.5 mg/l, however in other cases a poor AMR classification score does not correlate to proximity to the MIC boundary (Fig. 3, Table 2). Instead, they show a stronger correlation to a poor classification accuracy in the strain identification analyses (Fig. 2).

Analysis with the MX-Raman approach performed better than either of the two single-excitation analyses. It performed the best out of the three approaches in 68.75% of the classifications across the four antibiotics tested, and was the poorest in 5% classifications (PA21 for ciprofloxacin resistance, PA30 for tobramycin resistance, PA44 for imipenem resistance, and PA57 for ceftazidime resistance) (Fig. 3). The macro F1 scores of the MX-Raman analyses scored highest for each antibiotic. This is reflected in the scores for separated sensitive and resistant classifications, the MX-Raman analysis performed best for both categories for all antibiotics, aside from spectra classified sensitive to ciprofloxacin where the scores at 532 nm and using MX-Raman were both 0.97 (Table 3).

**Table 3:**
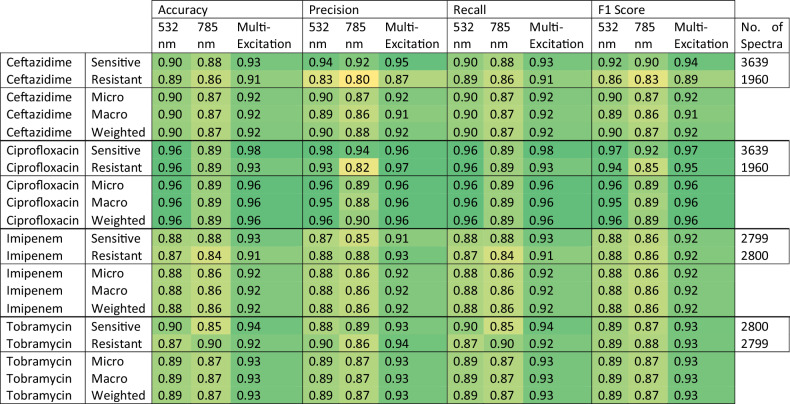
Antibiotic-Sensitivity SVM Classification Performances using Single-Excitation and Multi-Excitation Raman Spectroscopy

## Discussion

Using MX-Raman, we were able to accurately classify bacteria to the strain level and predict their antimicrobial resistance profiles, with minimal sample processing. A range of classification models were used to objectively evaluate the discriminative capabilities of the single- and multi-excitation approaches and determine which yields the highest classification performance. An SVM model was used to classify 20 *P. aeruginosa* clinical isolate strains with a 87% success rate (Table 1). Higher success rates were achieved when classifying the strains by their antimicrobial resistance profiles. Using an MX-Raman SVM model, successful classification of the strains’ resistance to four antibiotics was achieved with a macro-averaged F1 score ranging from 91% (ceftazidime) to 96% (ciprofloxacin) (Table 3).

The MX-Raman analysis consistently outperformed both analyses with either of the single excitations (Figs. 2, 3**)**. Where misclassifications occur in one of the analyses with either single-excitation, the MX-Raman approach compensated and decreased them. For example, PA55 is misclassified as PA30 and PA44 in the analysis with the single-excitation of 532 nm, and PA21 and PA56 in the analysis with the single-excitation of 785 nm, but this is corrected for in the MX-Raman analysis with a total classification accuracy of 76.8% (Fig. 2d). PA30 is misclassified as PA55, and PA58 in both single-excitation approaches, with classification accuracies of 42.1% and 52.5%. In this case, the error remained in the MX-Raman classification closer to the lower of the two values, 42.9%. There were no instances where MX-Raman analysis performed worse at strain identification than the analyses with either of the single-excitations (Fig. 2b–d, Table 1), and there were only four instances of MX-Raman performing worse in the AMR profiling analyses (Fig. 3). We envision that in practice, a resulting diagnostic software tool will take each of the analyses with each of three approaches into account, with appropriate and distinct weightings, as the single-excitation measurements need to be taken for the MX-Raman approach. This would ensure that a diagnosis could be reached with maximum confidence.

The best performing model selected for strain classification and AMR profiling was SVM, despite each model being tested independently for each antibiotic (Figs. 2, 3, Supplementary Table 1, Supplementary Table 3). The differences between the best performing models were marginal in some cases, e.g. SVM and PCA-GradBoost both performed at 0.85 for analysis with excitation at 532 nm (Supplementary Table 3) but SVM was selected for optimal performance across the three wavelength analyses. We expect that the growing capacity for computational modelling in aiding diagnosis in the healthcare sector will facilitate the operation of multiple analytical models within a single diagnostic tool, so that in the future AMR can be thoroughly characterised for any given isolate.

The models performed better when classifying strains’ resistance to ciprofloxacin than to other antibiotics (Table 3). We hypothesise that this is due to ciprofloxacin resistance being typically mediated by high expression of efflux pumps^29^, and the effect of these protein structures on the *P. aeruginosa* cell wall is large enough to affect the Raman spectral signatures of these strains. Conversely, imipenem resistance is mediated by carbapenemases present inside the cell^30^ where their expression may in some cases be too dilute to result in detectable changes in the Raman spectra of those strains, leading to a higher incidence of misclassification. Ceftazidime resistance can be mediated by both efflux pumps and ß-lactamases^31^, which may contribute to its lower F1 score of 91%. While these data are encouraging, further work is required to definitively link Raman spectral features to molecular components of bacteria.

An important consideration of this study was that bacterial replicates were cultured under uniform conditions to limit intra-strain variability in resulting Raman spectra. Despite this, a subset of strains display variation in their spectra, e.g. PA26, PA57, and PA68 (Fig. 1b). In the current work, this variation in spectra was related to sample fluorescence, which in turn could be caused by the production of pigments. In a diagnostic setting using direct analysis of patient samples, phenotypic variation in the pathogen is likely to be greatly amplified in response to a range of ongoing antimicrobial therapies, microbiomes, and variation in the patients’ mucus itself^32,33^.

This work also demonstrated the extent to which sample presentation for measurement can affect the data output, where a glass artefact present at 800 cm^−1^ for PA05 and PA10 spectra resulted in their partial misclassification under analysis with 532 nm excitation, which was then resolved by MX-Raman analysis (Figs. 1, 2b–d). This artefact was a result of a thin bacterial sample spread across the substrate, so the glass signal was detected beneath a compromised aluminium sputter coating. Further development of the technique for clinical samples will require either a minimum thickness of sample, a processing step to remove the 800 cm^−1^ glass artefact from the data output or use an aluminium support instead of an aluminium-coated glass slide for sample presentation.

Raman spectroscopy is an emerging technique as a microbial diagnostic and has been the subject of several recent reviews that hypothesise its use in the characterisation of AMR^21,34,35^. Work towards this application to date has focused on SERS, which improves sensitivity of Raman at the cost of additional sample preparation. In the clinic, this preparation is likely to include bacterial isolation and incubation, drastically increasing sample preparation time and negating the advantage of rapidity of Raman analysis. Raman spectroscopy techniques have been investigated as an alternative strategy for AST, measuring the differences between Raman signatures or pathogens that are sensitive and resistant to antibiotic treatments^36–39^. Thrift et al.^39^ used SERS to collect spectra of *P. aeruginosa and E. coli* subjected to a range of concentrations of gentamycin, from which a deep neural network could be trained. The deep learning analyses determined that the sensitivity of the bacteria to gentamycin could be predicted from SERS spectra taken after 10 mins exposure. Deuterium labelling is also used to measure changes to bacterial metabolism in response to antibiotic treatment, via Raman spectroscopy^40–42^. Both of these approaches reduce the time required to reach an accurate diagnosis and treatment decision; however, separation from the sample matrix and pathogen isolation is still required to achieve useful spectra from clinical samples. AMR characterisation has been studied by directly comparing the spectra of bacterial strains with different AMR profiles^16,43^, similar to the present study. In such cases, the AMR profiling is predicated on assigning test spectra to one of a defined library of bacterial strains, where the AMR profiles have been determined for those specific strains. These models are not necessarily able to predict the antimicrobial profiles of unknown bacterial species and strains, which are a critical avenue of future work. In this study, AMR profiling was correlated to 20 clinical isolates of the same bacterial species. With a substantially larger dataset of characterised *P. aeruginosa* isolates, an adaptive model capable of classifying the AMR profiles of unseen *P. aeruginosa* strains based on its Raman spectral profile may be possible.

Raman spectroscopy as a potential diagnostic tool for infection offers some advantages against other emerging rapid AST technologies. New microfluidics-based approaches offer rapid diagnostic outputs at the single cell level, that like Raman can be integrated into machine learning models^12,44^. Kandavalli et al developed a methodology that measures growth of mixed microbial populations in microfluidic chambers in the presence and absence of antibiotics, followed by identification by fluorescence in situ hybridisation (FISH)^44^. This provides a level of sensitivity that Raman spectroscopy is unable to reach, at a similar speed. However, this technology is currently limited by the need for specific FISH probe combinations for each species tested and may be limited in its ability to identify pathogens at the strain level. While Raman requires a database of spectra for each pathogen, it does not require the use of additional reagents. Microfluidic technologies and others such as MALDI-TOF also require sample preparation steps which are in principle not necessary for analysis by Raman spectroscopy, although this direct analysis aspect has not been investigated in this study.

This study used planktonic bacteria to generate the Raman library (Fig. 1), which allows for greater resolution of spectral features between strains at the cost of some verisimilitude for sputum samples of patients with respiratory infections. Bacterial respiratory infections such as in the cystic fibrosis lung or ventilator associated pneumonia typically grow as biofilms suspended within mucus^45,46^, so future work will require libraries of Raman data derived from *P. aeruginosa* biofilms, tested against clinical samples for rapid and direct identification. Additionally, this proof-of-concept study focuses solely on *P. aeruginosa*, where additional datasets must be raised in future to test the profiling capability of the MX-Raman methodology on other bacterial pathogens. This will likely include further optimisation of the data processing and analysis techniques to account for physiological differences in different bacterial species. different bacterial species. The challenges associated with retrieving bacterial Raman signal from complex clinical samples have previously been considered by Rusciano et al.^47^, who utilised photobleaching steps to reduce fluorescence of sputum samples, achieving classification of samples containing either *P. aeruginosa* or *S. aureus*. We hypothesise that MX-Raman will provide enhanced spectral ‘fingerprinting’ capability for microbial characterisation in complex samples such as these^13^. Additional optimisation will also be required to apply this methodology to direct clinical samples, as Raman signal arising from host factors may influence or interfere with pathogen identification and sample preparation procedures to concentrate the bacterial component of the sample will undermine the benefit of its rapidity.

This study provides a proof-of-concept for the use of MX-Raman as a tool to characterise the AMR profiles of *P. aeruginosa*. The methodology described uses direct analysis of bacterial samples, requiring minimal sample processing. Our approach has resulted in the use of multiple analytical models to classify bacteria, dependent on classification criteria (e.g. strain or resistance to a specific drug). We envision that this work could form the basis of a direct diagnostic tool for bacterial infection, acting as a rapid pre-screen ahead of confirmation of pathogen presence by conventional techniques. Several challenges must be addressed in future work ahead of the translation of this methodology, including its expansion to other bacterial species, integration with automated machine learning models, and optimisation for in situ analysis of sputum samples.

## Methods

### Bacteria and culture

We isolated twenty *Pseudomonas aeruginosa* clinical strains (Fig. 1) from expectorated sputum derived from people with cystic fibrosis, as previously described^48^. Briefly, sputum was treated with Mucolyse™ Sputum Digestant (Pro-Lab Diagnostics, UK) for 15 min at 37 °C. Confirmation of *P. aeruginosa* was carried out by growth of the isolates on cetrimide agar (Sigma-Aldrich, UK), and validated with multiplex PCR^48^. Sputum samples were collected for a previous study and all sampling protocols and procedures were approved by the UK NHS Research Ethics Committee (REC No: 08/H0502/126)^48^. Bacterial isolates were stored in 25% glycerol stocks at −80 °C. Bacterial cultures were grown in Mueller-Hinton broth (MHB) (Sigma-Aldrich, USA) for AST and in Lysogeny broth Miller (Formedium, UK) for Raman spectroscopy, both for 18 h at 37 °C, with shaking at 180 rpm.

### Antibiotic preparation and MIC testing

Minimum inhibitory concentration (MIC) tests were conducted in accordance with EUCAST guidelines. *P. aeruginosa* cultures were prepared to a concentration of 10^5^ CFU/ml in MHB and added to a twofold dilution series of ceftazidime, ciprofloxacin, imipenem, or tobramycin, ranging from 0.25 μg/ml to 256 μg/ml. Muller-Hinton broth 2 was used for tobramycin MIC assays. A stock solution of ciprofloxacin was prepared in 0.1 M HCl before suspension in MHB. Bacterial growth inhibition was measured using absorbance at 600 nm with a FLUOstar optima plate reader (BMG Labtech). At least three biological repeats were performed for each strain, and strains were designated ‘sensitive’ or ‘resistant’ according to EUCAST clinical breakpoints^49^. The intermediate classification ‘I’, representing strains susceptible to the antibiotic with increased exposure, was removed to simplify the classification criteria of the model, and strains were instead classified as ‘sensitive’. MIC testing was conducted with three technical repeats per antibiotic concentration, and at least three biological repeats per *P. aeruginosa* strain (Table 2, Supplementary Table 2).

### Raman microspectroscopy

Bacterial cultures were grown to a concentration of 10^8^ CFU/ml. Bacteria were washed twice by centrifugation (4000 g, 10 min) in ddH_2_O (W4502, Sigma-Aldrich). Concentrated bacteria were then resuspended in 250 μl ddH_2_O and dried onto an aluminium sputter-coated microscope glass slide (1 µm thickness, SSAL-01000-Q5, Angstrom Engineering, Canada) in air with gentle heating (40 °C).

Raman microspectroscopy experiments were conducted using a Renishaw InVia Raman microscope (Renishaw, UK), with a Leica DM 2500-M bright field microscope and an automated 100 nm-encoded *XYZ* stage. The samples were excited using a 100 mW DPSS 532 nm laser and a 100 mW near infrared point source diode laser at 785 nm (Renishaw plc.) directed through a Nikon 50× air objective (NA = 0.5). This gives a diffraction limited spot size of ~740 nm and 1.1 µm, respectively. The signal was collected after a Rayleigh edge filter appropriate to each excitation wavelength, and a diffraction grating (532 nm: 1800 L/mm, 785 nm: 1200 L/mm) that dispersed the Raman-scattered light onto a Peltier-cooled CCD (1024 pixels × 256 pixels). The peak at 520 cm^−1^ from a silicon wafer was used to calibrate the Raman wavenumber axis and was also used to calculate the spectral resolution of the spectrometer using 532 nm and 785 nm excitation wavelengths. The half-width half-maximum spectral resolution using 532 nm excitation was measured to be 2.96 cm^−1^, and using 785 nm excitation was measured to be 2.54 cm^−1^. For bacterial samples, spectra were typically acquired over three accumulations of 5 s each, using ~0.9 mW power for acquisitions using the 532 nm laser, and ~6.3 mW power for acquisitions using the 785 nm laser. Five biological repeats of each bacterial strain were used, fifty-six spectra were acquired for each biological repeat for each laser wavelength tested. The z-position was determined using a depth series analysis on the dried sample and selecting the z-position that produced the strongest peak intensity per sample. Measurements made using the two laser wavelengths were positioned at the same or similar x-y locations, e.g. at a position of similar sample thickness and distance from the sample edge if not at the exact same location.

### Data pre-processing and statistical analysis

Data preprocessing and statistical analysis were conducted in Renishaw WiRe 5.5.0 and Python v3.12.0 using the packages SciPy v1.13.0^50^, RamanSPy v0.2.7^26^, and scikit-learn v1.4.2^27^. In this study, raw spectra were divided into two datasets according to their excitation wavelength and pre-processed independently of each other. A third multi-excitation dataset was then derived after pre-processing of the two single-excitation datasets.

### Quality control screening

Prior to spectral pre-processing, all spectra showing effects of saturation, burning, or defocusing were identified and excluded from further analysis. To allow for direct comparison between all datasets, the number of spectra per biological replicate was standardised, and screening was performed on corresponding pairs of spectra. That is, exclusion of a 532 nm excitation spectrum from one biological replicate resulted in the screening of the corresponding 785 nm excitation spectrum acquired at the same position on the same biological replicate, and vice versa. This resulted in a total of 20 strains, with 280 spectra per strain, except for PA26 with a total of 279 spectra, for each dataset.

### Spectral pre-processing

Spectra remaining after quality screening were pre-processed to remove spectral interferents and corrupting artefacts. Pre-processing is frequently deemed a necessity for downstream analysis, as changes in experimental conditions may cause unwanted spectral contributions. These in turn, may mask the subtle but relevant underlying biological differences between highly similar spectra, reducing the efficacy of the final statistical model. Here spectral pre-processing was applied to correct for errors occurring due to the measurement instrument (i.e. cosmic noise, Gaussian noise, and intensity fluctuations) and the sample itself (i.e. fluorescence background). The order of pre-processing methods applied followed the pipeline illustrated by Guo et al.^28^.

Firstly, cosmic ray removal was employed using the nearest neighbour algorithm in *Wire 5.5*. Following this, spectra were smoothed using a second-order Savitsky-Golay filter with a window length of 5 to reduce Gaussian noise (via preprocessing.denoise.SavGol() function from *RamanSPy v0.2.726*^50^). The resultant spectra were then interpolated onto a common wavenumber axis with a step-size of 1 cm^−1^ using a radial basis interpolation function (using interpolate.Rbf() from *SciPy v1.13.025*^50^). Baseline subtraction via the asymmetric least squares algorithm (preprocessing.baseline.ALS() from *RamanSPy v0.2.726*^51^) with the parameters, *lambda*, and *p* set to 5000, and 0.01, respectively, was then applied to reduce background fluorescence intensity. Next a minimum intensity shift was applied to set the minimum intensity of each spectrum to zero and ensure all intensity values were non-negative. Finally, spectra were truncated to the fingerprint region, 600 to 1700 cm^−1^ (via preprocessing.misc.Cropper() from *RamanSPy v0.2.726*^51^), before intensity normalisation via area under the curve scaling (using the preprocessing.normalise.AUC() function from *RamanSPy v0.2.726*^51^).

To ensure correct implementation of each pre-processing operation, spectra were visually inspected following each applied procedure for artificial artefacts arising due to misuse of correcting algorithms. Supplementary Fig. 3 illustrates an example plot following the pre-processing pipeline of a spectral map obtained from a biological replicate of the strain *PA08* with a 532 nm excitation laser.

### Multi-excitation dataset construction

Following spectral pre-processing, a third multi-excitation dataset was obtained by concatenating each 532 nm excitation spectrum with the corresponding 785 nm excitation spectrum of the same position on the same biological replicate. This resulted in a total of three spectral datasets—two single-excitation (532 nm, and 785 nm) and one multi-excitation dataset—that were each used independently for the five bacterial characterisation tasks.

### Machine learning and statistical modelling

The applied machine learning modelling approach was developed identically for both the strain and the four antibiotic-sensitivity classification tasks. For ease of notation, in what follows, let a class denote either the strain or antibiotic-sensitivity, let a classifier denote a classification algorithm (potentially combined with an internal dimension reduction algorithm), and let a model denote a classifier trained on a specific dataset (i.e. either a single- or multi-excitation dataset).

To compare the discriminative capabilities of the single- and multi-excitation techniques and determine the most effective analysis model for each characterisation task, nine classifiers were trained on each of the three datasets—for a total of *27* models. The classification algorithms: *linear discriminant analysis* (*LDA*) with an internal *principal components analysis* (*PCA*) dimension reduction (*PCA-LDA*), *partial-least-squares discriminant analysis* (*PLS-DA*), *random forest* (*RF*), *support vector machine* (*SVM*), *SVM* with an internal *PCA* dimension reduction (*PCA-SVM*), *gradient boosting* (*GradBoost*), *GradBoost* with an internal *PCA* dimension reduction (*PCA-GradBoost*), *logistic regression* (*LogReg*), and *Log-Reg* with an internal *PCA* dimension reduction (*PCA-LogReg*) were selected to represent a range of parametric and non-parametric, ensemble, regularised and latent variable learners, and models with linear and non-linear decision boundaries. For a few classification algorithms considered susceptible to highly correlated or noisy features (i.e. *LDA*, *SVM*, *GradBoost*, and *LogReg*), *PCA* was applied internally to reduce collinearity and size of the feature space. Due to the long computational time and to avoid risk of over-fitting, an alternative dimension reduction method was also applied to the classifiers *LogReg*, *GradBoost* and *RF*, via the function *feature_selection.SelectKBest()* from *scikit-learn v1.4.2*^52^. This method selects the top *k* features from the spectral dataset by evaluating each feature independently with the target variable using the ANOVA F-value test. Finally, where it was deemed appropriate, features of the training sets were standardised to remove the mean (i.e. set to zero) and scale to unit variance using the function *preprocessing.StandardScalar()* from *scikit-learn v1.4.2*^52^. Details on the precise pipeline and implementation used for each classifier are provided in Supplementary Table 4.

### Cross-validation, performance metrics and hyperparameter optimisation

To benchmark the performance of each model a leave-one-biological-replicate out cross-validation was conducted. By splitting spectral datasets according to replicate information, each biological replicate was treated as an independent sample. In this way, all spectra from a single biological replicate were used either entirely for training or testing, thereby avoiding data leakage within the model - which may otherwise occur due to high correlation between spectra from the same replicate. The leave-one-biological-replicate-out cross-validation was selected for model testing, over the more common fold splitting method to maximise the available training data.

In addition to an outer cross-validation strategy to estimate model generalisability, an internal stratified *5-*biological-replicate-fold cross-validation procedure was implemented to optimise the hyperparameters of the algorithms in each analysis pipeline. The investigated hyperparameters for each classifier are provided in Supplementary Table 4.

Unlike the model parameters which are tuned during training of a machine learning model, hyperparameters are a set of configurations that control the behaviour of the learning algorithm and are set before training. As these variables can greatly influence the model’s performance, hyperparameters should be optimised using validation sets to improve the model’s effectiveness on unseen data. Like testing, hyperparameter tuning can be achieved using a cross-validation strategy that splits the training data (from the train-test splitting) into an inner training and inner validation set. For each combination of hyperparameters, a classifier is trained on the inner training set, and its performance is measured on the validation set. The combination of hyperparameters that yield the highest result on the validation set is then selected. Finally, a new machine is trained on the outer training set (i.e. both the inner training and inner validation set) with the selected hyperparameters, and the final testing score is evaluated on the testing set.

As with the sampling strategy used for testing, the cross-validation strategy for hyperparameter tuning was selected to respect spectral groups regarding biological replicates (i.e. splitting did not occur between spectra from the same sample/biological replicate). A stratified 5-biological-replicate-fold operation was selected to act internally within the leave-one-biological-replicate-out testing cross-validation to optimise the algorithms’ hyperparameters. Through this procedure, the training data was split into five subsets each consisting of exactly one biological replicate of each of the 20 strains in the strain characterisation problem (save for a single fold be missing the strain excluded for testing), and a proportional distribution of sensitive and resistant labelled samples in the antibiotic-sensitivity characterisation tasks. This precise method was selected to best fit the properties of the three datasets - that is, five biological replicates per strain and an imbalanced class structure for the antibiotic-sensitivity tasks.

The selected performance metric for measuring the power of each hyperparameter combination greatly influences which variables are used by the final model. Commonly used metrics include accuracy, precision, recall, and F1 score. While accuracy simply measures the proportion of correct predictions, it can be misleading in cases of class imbalance (as in the case of the antibiotic-sensitivity tasks), where the model may achieve a high accuracy by mostly predicting the majority class. The accuracy for each class in terms of the number of true positives (TP), true negatives (TN), false positives (FP), and false negatives (FN) is provided below (Equation 1).

1 $${Accuracy}=\frac{{TP}+{TN}}{{TP}+{TN}+{FP}+{FN}}$$

In contrast, precision (Equation 2) and recall (Equation 3) provide more nuanced insight into how well the model identifies positive instances, with precision focusing on how many predicted positives are correct, and recall on how many actual positives are captured.

2 $${Precision}=\frac{{TP}}{{TP}+{FP}}$$

3 $${Recall}=\frac{{TP}}{{TP}+{FN}}$$

Finally, the F1 score (Equation 4) balances these two by taking their harmonic mean, thus reflecting the trade-off between precision and recall. Here F1 score was selected to be optimised to ensure that neither false positives nor false negatives were disproportionately ignored, thereby leading to a more robust model performance.

4 $${F}_{1}=2\times \frac{{Precision}\times {Recall}}{{Precision}+{Recall}}=\frac{2\times {TP}}{2\times {TP}+{FP}+{FN}}$$

In addition to selecting an appropriate metric, choosing a suitable averaging method is also crucial in multi-class (i.e. strain identification) and imbalanced classification (i.e. sensitivity detection) problems, as the precision, recall, and F1 are scored per class. The three main types of averaging are macro, micro, and weighted. Micro-averaging aggregates the contributions of all classes to compute a global metric, which can bias the score toward the majority class. Weighted averaging, on the other hand, considers the number of instances (i.e. spectra) per class, but similarly favours classes with more instances. In contrast, macro-averaging calculates the metric (e.g. F1 score) independently for each class then takes the average, giving equal weight to all classes regardless of their frequency. By equally valuing performances across all classes, macro-averaging helps select hyperparameters that lead to a more balanced and fairer model. The macro-averaged F1 score was, therefore, selected as the metric for hyperparameter tuning to aid in the multi-class strain characterisation problem and the imbalanced antibiotic-sensitivity tasks.

To summarise, a leave-one-biological-replicate out cross-validation was applied to evaluate model performance, and a stratified *5-*biological-replicate-fold cross-validation was applied for hyperparameter optimisation using the macro-averaged F1 score as the validating metric.

For each characterisation task the highest performing classifier across the three excitation approaches was selected and investigated for further analysis. All previously described metrics (i.e. accuracy, precision, recall, and F1) and their associated averages (i.e. micro, macro, weighted) were recorded for each biological replicate in the testing set. The macro adjusted F1 score (i.e. the macro-averaged F1 minus the standard deviation of F1 scores across classes), was chosen to evaluate the highest performing model, and thereby select the model that balances both high overall performance and low variability. The equation for the macro adjusted F1 score for an *N*-class classification problem in terms of the F1 score for each class i, F_1_(i) is provided below (Equation 5).

5 $${Adjusted}\,F1=\frac{1}{N}\mathop{\sum }\limits_{i=1}^{N}{F}_{1}\left(i\right)-\sqrt{\frac{1}{N}\mathop{\sum }\limits_{i=1}^{N}{\left({F}_{1}\left(i\right)-\frac{1}{N}\mathop{\sum }\limits_{j=1}^{N}{F}_{1}\left(j\right)\right)}^{2}2}$$

## Supplementary information

Supplementary Figures

## Data Availability

Raman spectroscopy datasets generated in this study have been deposited onto the University of Southampton data repository, Pure, with UUID: 1db35a15-be53-4863-a5fb-d29bf12cb078. Data will be made available on request.
