# Supplementary material for: Identification and antimicrobial resistance profiling of Pseudomonas aeruginosa using multi-excitation Raman spectroscopy and computational analytics
Source: NPJ Antimicrob Resist. 2025 Aug 25;3:74. doi: 10.1038/s44259-025-00141-z (PMC12378394; doi:10.1038/s44259-025-00141-z)
Supplement: Supplementary file 1 — Supplementary Figures [file 44259_2025_141_MOESM1_ESM.pdf]

# Supplementary Figures

## *Supplementary Figure 1: Principal Component Analysis Cluster Plots*

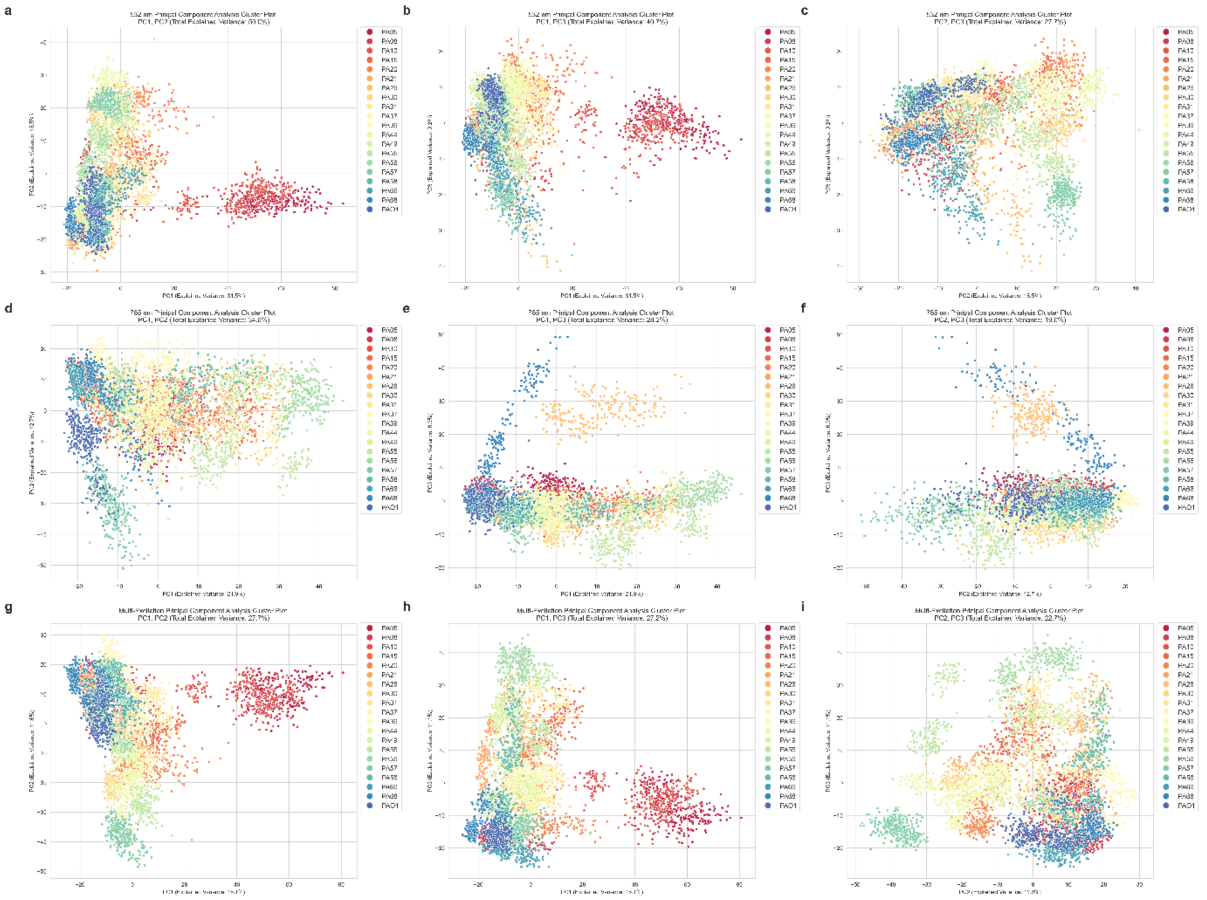

Supplementary Figure 11 **Principal Component Analysis Cluster Plots for 20 *Pseudomonas aeruginosa* Clinical Isolates obtained using Single-Excitation and Multi-Excitation Raman Spectroscopy.** The spectral clusters of each strain were visualised for each of the three preprocessed spectral datasets: (a) – (c) 532 nm, (d) - (f) 785 nm, and (g) – (i) Multi-excitation Raman, using the first three principal components (PC1, PC2, and PC3).

***Supplementary Figure 2: Principal Component Analysis Loadings Plots for 20 Pseudomonas aeruginosa Clinical Isolates obtained using Single-Excitation and Multi-Excitation Raman Spectroscopy***

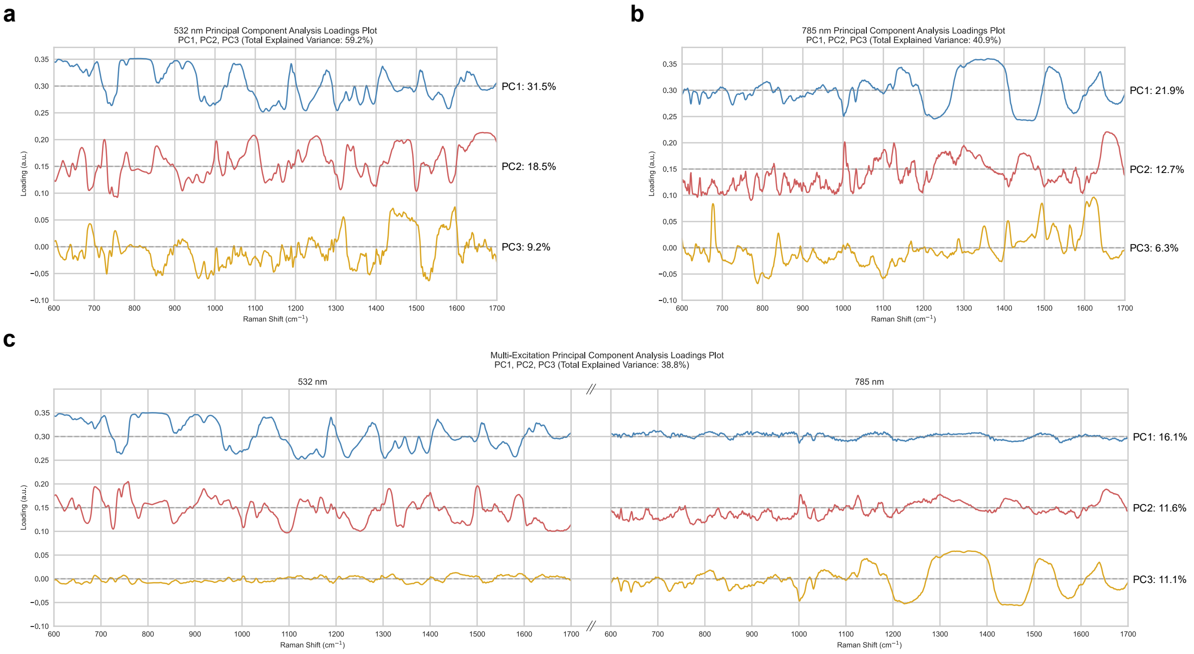


Supplementary Figure 22 **Principal Component Analysis Loadings Plots for 20 *Pseudomonas aeruginosa* Clinical Isolates obtained using Single-Excitation and Multi-Excitation Raman Spectroscopy.** The loadings of the first three principal components (PC1, PC2, and PC3) for each of the three pre-processed spectral datasets: (a) 532 nm, (b) 785 nm, and (c) Multi-excitation Raman, were investigated to evaluate the wavenumber features that contributed the highest variance in each dataset.

## *Supplementary Figure 3: Spectral Pre-Processing Pipeline*

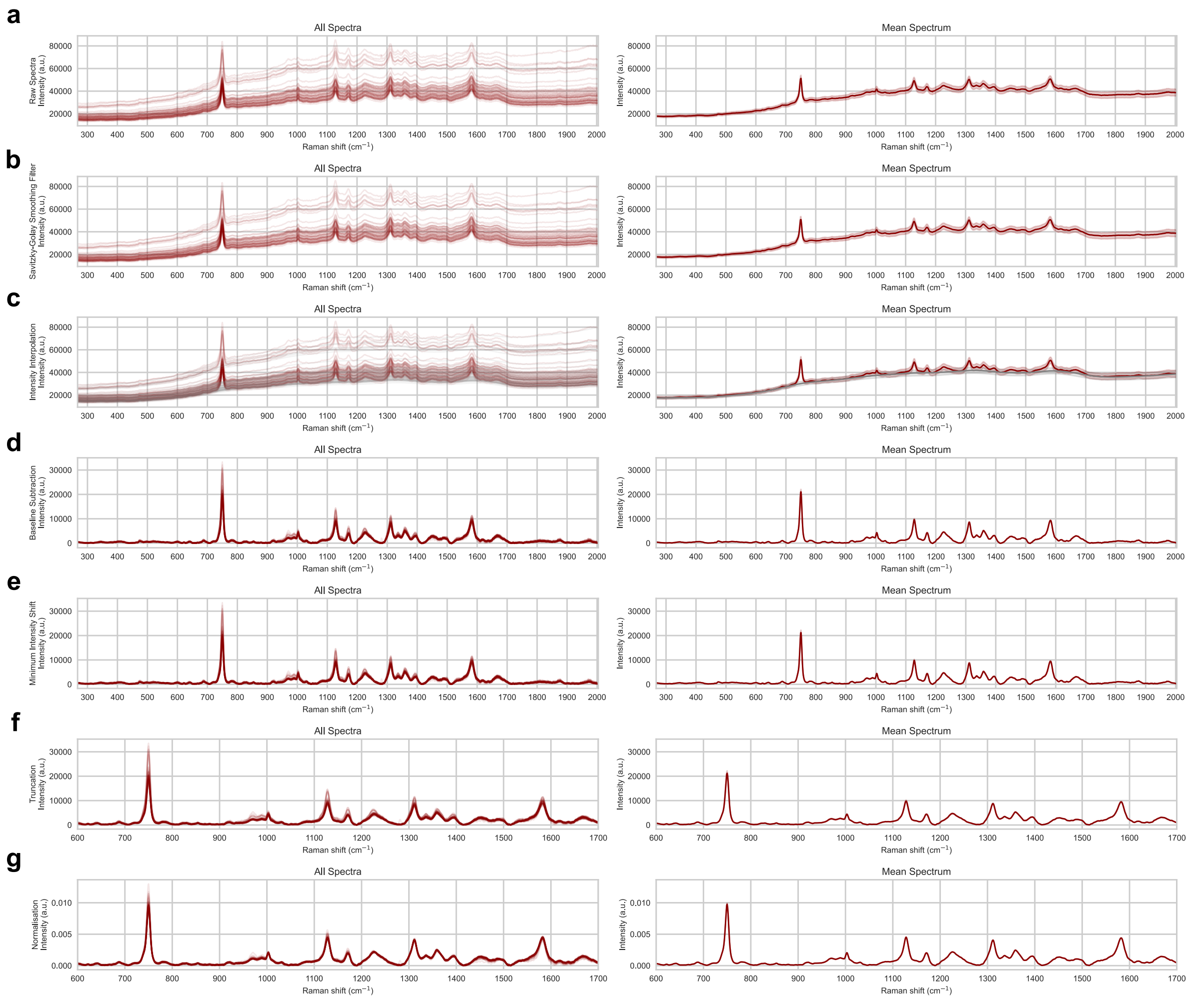


Supplementary Figure 3 **Spectral Pre-Processing Pipeline.** Prior to multivariate analysis, spectra were pre-processed to remove interferents and corrupting artefacts. To ensure correct implementation of each correcting algorithm, spectra were visualised before and after each pre-processing operation. In addition to the mean spectrum and standard deviation (RIGHT), all spectra from a single spectral map were visualised to detect outliers occurring within the multi-acquisition measurement (LEFT). An identical pre-processing pipeline was applied to all spectra in the bacterial library across both strains and excitation wavelengths. The applied pipeline to transforming the (a) raw spectra of each spectral map consisted of (b) a Savitsky-Golay smoothing filter, (c) a wavenumber interpolation, (d) a baseline subtraction via the asymmetric least squares algorithm, (e) a non-negative-intensity shift, (f) a truncation to the fingerprint region (600-1700 cm-1), and (g) a normalisation via an area under the curve scaling.

***Supplementary Figure 4: Per Strain Classification Performances for Strain Identification of 20 Pseudomonas aeruginosa clinical isolates using Single- and Multi-excitation Raman Spectroscopy***

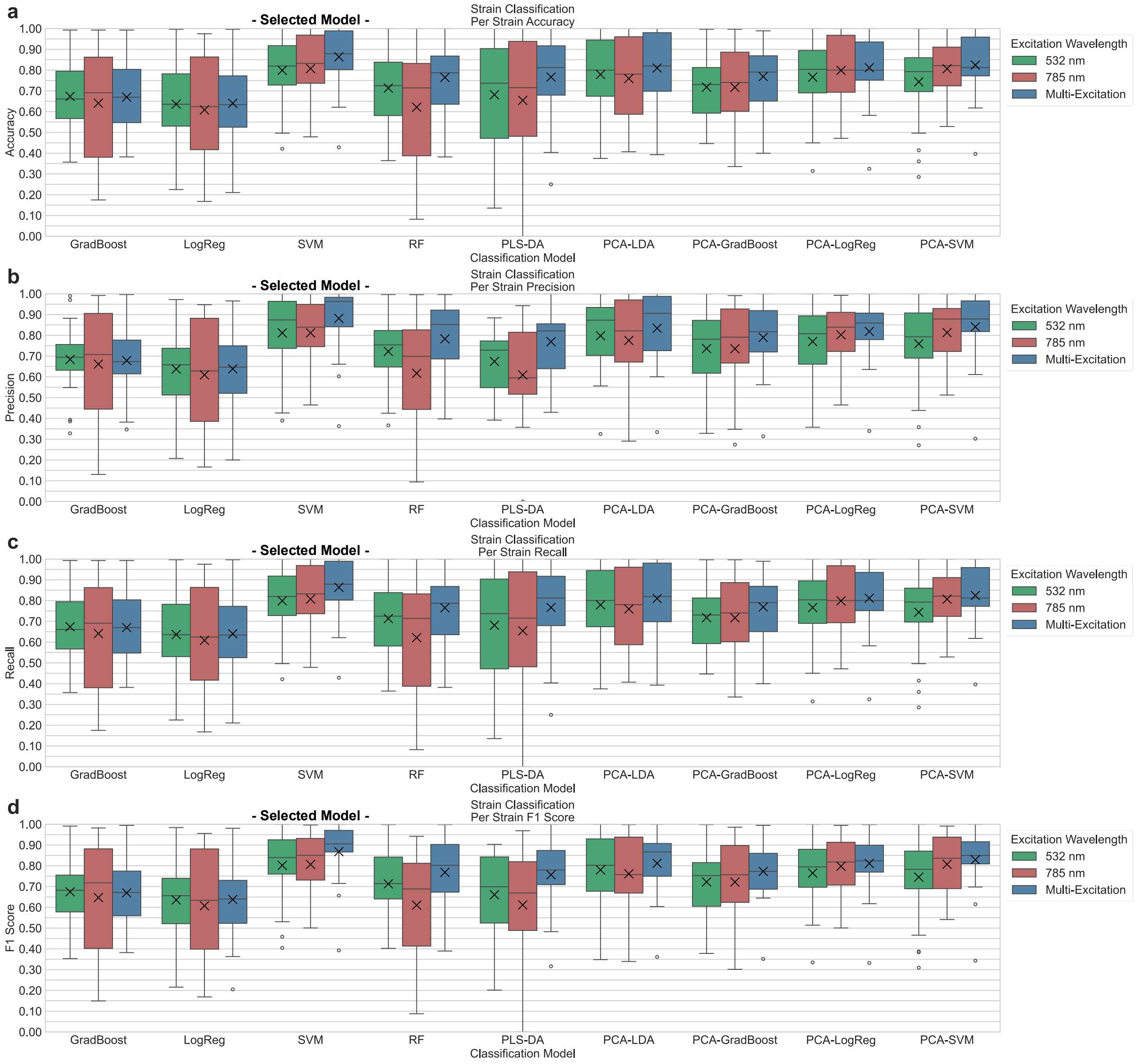


Supplementary Figure 4 **Per Strain Classification Performances for Strain Identification of 20 *Pseudomonas aeruginosa* clinical isolates using Single- and Multi-excitation Raman Spectroscopy.** Nine machine learning classifiers (GradBoost, LogReg, SVM, RF, PLS-DA, PCA-LDA, PCA-GradBoost, PCA- LogReg, PCA-SVM) were applied to each of the three Raman spectral datasets (532 nm, 785 nm, and multi-excitation) for strain identification of 20 *Pseudomonas aeruginosa* clinical isolates. The highest performing classier was selected based on the adjusted F1 Score calculated as the macro mean F1 score (indicated by an 'X' in (d)) minus the standard deviation in scores across strains. To further validate model performance, the per strain (a) accuracy, (b) precision and (c) recall were also compared across excitation approaches and classifiers. Across all excitation approaches, and investigated metrics, the SVM classifier was identified as the highest performing model and selected for further investigation.

# Supplementary Tables

## *Supplementary Table 1: Strain Identification Model Selection*

Supplementary Table 1 **Strain identification Model Selection.** Nine machine learning classifiers (GradBoost, LogReg, SVM, RF, PLS-DA, PCA-LDA, PCA-GradBoost, PCA- LogReg, PCA-SVM) were applied to each of the three Raman spectral datasets (532 nm, 785 nm, and multi-excitation) for strain identification of 20 *Pseudomonas aeruginosa* clinical isolates. The highest performing classier was selected based on the adjusted F1 score - calculated as the macro mean F1 score minus the standard deviation across the per strain F1 scores. As a high adjusted F1 score indicates a high overall performance and low variability across strain classes, each classifier was ranked in order of increasing adjusted F1 score, and the lowest rank model was selected. To further validate model performance, the macro-averaged accuracy, precision, and recall were also compared across all excitation approaches and classifiers. Across all excitation approaches, and investigated metrics, the SVM classifier was identified as the highest performing model and selected for further investigation.

| | Accuracy | | | Precision | | | Recall | | | F1 Score | | | Adjusted F1 Score | | | |
|---|---|---|---|---|---|---|---|---|---|---|---|---|---|---|---|---|
| | 532 nm | 785 nm | Multi-Excitation | 532 nm | 785 nm | Multi-Excitation | 532 nm | 785 nm | Multi-Excitation | 532 nm | 785 nm | Multi-Excitation | 532 nm | 785 nm | Multi-Excitation | Rank |
| GradBoost | 0.67 | 0.64 | 0.67 | 0.68 | 0.66 | 0.68 | 0.67 | 0.64 | 0.67 | 0.67 | 0.65 | 0.67 | 0.50 | 0.37 | 0.49 | 7 |
| LogReg | 0.64 | 0.61 | 0.64 | 0.64 | 0.61 | 0.64 | 0.64 | 0.61 | 0.64 | 0.64 | 0.61 | 0.64 | 0.45 | 0.34 | 0.45 | 9 |
| SVM | 0.80 | 0.81 | 0.86 | 0.81 | 0.81 | 0.88 | 0.80 | 0.81 | 0.86 | 0.80 | 0.81 | 0.87 | 0.63 | 0.64 | 0.72 | 1 |
| RF | 0.71 | 0.62 | 0.76 | 0.72 | 0.62 | 0.78 | 0.71 | 0.62 | 0.76 | 0.71 | 0.61 | 0.77 | 0.55 | 0.33 | 0.60 | 6 |
| PLS-DA | 0.68 | 0.65 | 0.77 | 0.67 | 0.61 | 0.77 | 0.68 | 0.65 | 0.77 | 0.66 | 0.61 | 0.76 | 0.47 | 0.34 | 0.59 | 8 |
| PCA-LDA | 0.78 | 0.76 | 0.81 | 0.80 | 0.78 | 0.83 | 0.78 | 0.76 | 0.81 | 0.78 | 0.76 | 0.81 | 0.61 | 0.56 | 0.66 | 4 |
| PCA-GradBoost | 0.72 | 0.72 | 0.77 | 0.74 | 0.74 | 0.79 | 0.72 | 0.72 | 0.77 | 0.72 | 0.72 | 0.77 | 0.56 | 0.51 | 0.63 | 5 |
| PCA-LogReg | 0.77 | 0.80 | 0.81 | 0.77 | 0.80 | 0.82 | 0.77 | 0.80 | 0.81 | 0.77 | 0.80 | 0.81 | 0.59 | 0.65 | 0.66 | 2 |
| PCA-SVM | 0.74 | 0.81 | 0.82 | 0.76 | 0.81 | 0.84 | 0.74 | 0.81 | 0.82 | 0.75 | 0.81 | 0.83 | 0.54 | 0.66 | 0.68 | 3 |

***Supplementary Table 2: Raw data for minimum inhibitory concentration tests for 20 clinical isolates of Pseudomonas aeruginosa to the antibiotics: ceftazidime, ciprofloxacin, imipenem, and tobramycin (units mg/l).***

Supplementary Table 2 **Raw data for minimum inhibitory concentration tests for 20 clinical isolates of *Pseudomonas aeruginosa* to the antibiotics: ceftazidime, ciprofloxacin, imipenem, and tobramycin (units mg/l).**

| | Tobramycin MIC | | | Average | Ceftazidime MIC | | | | Average | Ciprofloxacin MIC | | | Average | Imipenem MIC | | | | Average |
|---|---|---|---|---|---|---|---|---|---|---|---|---|---|---|---|---|---|---|
| PAO1 | 0.50 | 0.25 | 0.25 | 0.33 | 4.00 | 4.00 | 2.00 | | 3.33 | 0.50 | 0.50 | 1.00 | 0.67 | 2.00 | 2.00 | 4.00 | | 2.67 |
| PA05 | 0.25 | 0.25 | 0.25 | 0.25 | 1.00 | 1.00 | 1.00 | | 1.00 | 0.25 | 0.50 | 0.50 | 0.42 | 1.00 | 1.00 | 2.00 | | 1.33 |
| PA08 | 1.00 | 1.00 | 0.50 | 0.83 | 64.00 | 64.00 | 64.00 | | 64.00 | 0.25 | 0.25 | 0.25 | 0.25 | 1.00 | 1.00 | 0.50 | | 0.83 |
| PA10 | 0.50 | 0.50 | 0.25 | 0.42 | 1.00 | 4.00 | 2.00 | | 2.33 | 0.25 | 0.50 | 0.25 | 0.33 | 0.50 | 1.00 | 0.50 | | 0.67 |
| PA15 | 4.00 | 2.00 | 2.00 | 2.67 | 1.00 | 0.50 | 1.00 | | 0.83 | 0.50 | 0.50 | 0.50 | 0.50 | 1.00 | 1.00 | 1.00 | | 1.00 |
| PA20 | 0.50 | 0.50 | 0.25 | 0.42 | 0.50 | 0.50 | 1.00 | | 0.67 | 0.25 | 0.50 | 1.00 | 0.58 | 2.00 | 0.50 | 1.00 | | 1.17 |
| PA21 | 0.50 | 1.00 | 0.25 | 0.58 | 64.00 | 32.00 | 32.00 | | 42.67 | 0.25 | 0.25 | 0.25 | 0.25 | 16.00 | 16.00 | 32.00 | | 21.33 |
| PA26 | 1.00 | 0.50 | 0.50 | 0.67 | 256.00 | 256.00 | 256.00 | | 256.00 | 0.50 | 1.00 | 0.50 | 0.67 | 16.00 | 32.00 | 32.00 | | 26.67 |
| PA30 | 0.25 | 0.25 | 0.25 | 0.25 | 16.00 | 4.00 | 8.00 | 2.00 | 7.50 | 0.50 | 1.00 | 0.50 | 0.67 | 8.00 | 16.00 | 4.00 | | 9.33 |
| PA31 | 4.00 | 1.00 | 2.00 | 2.33 | 128.00 | 16.00 | 64.00 | 32.00 | 60.00 | 0.25 | 0.25 | 0.50 | 0.33 | 4.00 | 16.00 | 2.00 | 8.00 | 7.50 |
| PA37 | 0.50 | 0.50 | 0.50 | 0.50 | 64.00 | 64.00 | 128.00 | | 85.33 | 1.00 | 1.00 | 1.00 | 1.00 | 1.00 | 8.00 | 2.00 | 4.00 | 3.75 |
| PA39 | 0.50 | 0.25 | 1.00 | 0.58 | 4.00 | 4.00 | 4.00 | | 4.00 | 0.25 | 0.50 | 1.00 | 0.58 | 8.00 | 2.00 | 4.00 | | 4.67 |
| PA44 | 64.00 | 128.00 | 64.00 | 85.33 | 32.00 | 32.00 | 4.00 | | 22.67 | 0.50 | 1.00 | 1.00 | 0.83 | 4.00 | 4.00 | 2.00 | | 3.33 |
| PA49 | 64.00 | 32.00 | 32.00 | 42.67 | 64.00 | 64.00 | 64.00 | | 64.00 | 0.50 | 0.50 | 1.00 | 0.67 | 16.00 | 8.00 | 32.00 | | 18.67 |
| PA55 | 256.00 | 128.00 | 256.00 | 213.33 | 8.00 | 4.00 | 32.00 | 64.00 | 27.00 | 0.50 | 1.00 | 1.00 | 0.83 | 2.00 | 1.00 | 1.00 | | 1.33 |
| PA56 | 64.00 | 64.00 | 64.00 | 64.00 | 128.00 | 32.00 | 64.00 | | 74.67 | 0.50 | 1.00 | 2.00 | 1.17 | 4.00 | 8.00 | 8.00 | | 6.67 |

| | | | | | | | | | | | | | | | | | |
|---|---|---|---|---|---|---|---|---|---|---|---|---|---|---|---|---|---|
| PA57 | 32.00 | 16.00 | 32.00 | 26.67 | 64.00 | 32.00 | 64.00 | | 53.33 | 0.50 | 0.25 | 1.00 | 0.58 | 4.00 | 4.00 | 8.00 | 5.33 |
| PA58 | 32.00 | 32.00 | 32.00 | 32.00 | 32.00 | 32.00 | 64.00 | 128.00 | 64.00 | 0.25 | 0.50 | 1.00 | 0.58 | 8.00 | 16.00 | 8.00 | 10.67 |
| PA66 | 64.00 | 64.00 | 64.00 | 64.00 | 64.00 | 64.00 | 64.00 | | 64.00 | 0.50 | 1.00 | 2.00 | 1.17 | 8.00 | 16.00 | 8.00 | 10.67 |
| PA68 | 16.00 | 32.00 | 32.00 | 26.67 | 64.00 | 64.00 | 64.00 | | 64.00 | 0.25 | 0.50 | 0.50 | 0.42 | 1.00 | 2.00 | 2.00 | 1.67 |

## *Supplementary Table 3: Antibiotic Sensitivity Profiling Model Selection*

Supplementary Table 3 **Antibiotic Sensitivity Profiling Model Selection.** Nine machine learning classifiers (GradBoost, LogReg, SVM, RF, PLS-DA, PCA-LDA, PCA-GradBoost, PCA- LogReg, PCA-SVM) were applied to each of the three Raman spectral datasets (532 nm, 785 nm, and multi-excitation) to determine the sensitivities of 20 *Pseudomonas aeruginosa* clinical isolates to the antibiotics: ceftazidime, ciprofloxacin, imipenem, and tobramycin. The highest performing classier for each antibiotic-sensitivity characterisation task was selected based on the adjusted F1 score - calculated as the macro mean F1 score minus the standard deviation across the per strain F1 scores. As a high adjusted F1 score indicates a high overall performance and low variability across strain classes, each classifier was ranked in order of increasing adjusted F1 score, and the lowest rank model was selected. To further validate model performance, the macro-averaged accuracy, precision, and recall were also compared across all antibiotic tasks, excitation approaches and classifiers. Across all antibiotics, excitation approaches, and investigated metrics, the SVM classifier was identified as the highest performing model.

| | | Accuracy | | | Precision | | | Recall | | | F1 Score | | | Adjusted F1 Score | | | |
|---|---|---|---|---|---|---|---|---|---|---|---|---|---|---|---|---|---|
| | | 532 nm | 785 nm | Multi-Excitation | 532 nm | 785 nm | Multi-Excitation | 532 nm | 785 nm | Multi-Excitation | 532 nm | 785 nm | Multi-Excitation | 532 nm | 785 nm | Multi-Excitation | Rank |
| Ceftazidime | GradBoost | 0.87 | 0.76 | 0.87 | 0.87 | 0.76 | 0.87 | 0.87 | 0.76 | 0.87 | 0.87 | 0.76 | 0.87 | 0.81 | 0.65 | 0.82 | 6 |
| Ceftazidime | LogReg | 0.84 | 0.67 | 0.84 | 0.84 | 0.68 | 0.85 | 0.84 | 0.67 | 0.84 | 0.84 | 0.67 | 0.84 | 0.77 | 0.51 | 0.78 | 8 |
| Ceftazidime | SVM | 0.90 | 0.87 | 0.92 | 0.89 | 0.86 | 0.91 | 0.90 | 0.87 | 0.92 | 0.89 | 0.86 | 0.91 | 0.85 | 0.81 | 0.88 | 1 |
| Ceftazidime | RF | 0.88 | 0.79 | 0.89 | 0.88 | 0.79 | 0.89 | 0.88 | 0.79 | 0.89 | 0.88 | 0.79 | 0.89 | 0.83 | 0.69 | 0.84 | 4 |
| Ceftazidime | PLS-DA | 0.87 | 0.80 | 0.89 | 0.88 | 0.80 | 0.90 | 0.87 | 0.80 | 0.89 | 0.87 | 0.80 | 0.89 | 0.82 | 0.71 | 0.85 | 2 |
| Ceftazidime | PCA-LDA | 0.80 | 0.73 | 0.81 | 0.81 | 0.73 | 0.82 | 0.80 | 0.73 | 0.81 | 0.81 | 0.73 | 0.81 | 0.72 | 0.62 | 0.72 | 9 |
| Ceftazidime | PCA-GradBoost | 0.89 | 0.79 | 0.87 | 0.89 | 0.80 | 0.88 | 0.89 | 0.79 | 0.87 | 0.89 | 0.80 | 0.88 | 0.85 | 0.71 | 0.82 | 3 |
| Ceftazidime | PCA-LogReg | 0.86 | 0.79 | 0.81 | 0.84 | 0.77 | 0.79 | 0.86 | 0.79 | 0.81 | 0.85 | 0.77 | 0.80 | 0.79 | 0.70 | 0.73 | 7 |
| Ceftazidime | PCA-SVM | 0.85 | 0.85 | 0.88 | 0.84 | 0.83 | 0.87 | 0.85 | 0.85 | 0.88 | 0.84 | 0.83 | 0.87 | 0.79 | 0.78 | 0.82 | 5 |
| Ciprofloxacin | GradBoost | 0.85 | 0.80 | 0.86 | 0.88 | 0.81 | 0.88 | 0.85 | 0.80 | 0.86 | 0.86 | 0.80 | 0.87 | 0.80 | 0.72 | 0.80 | 6 |
| Ciprofloxacin | LogReg | 0.74 | 0.66 | 0.75 | 0.76 | 0.67 | 0.77 | 0.74 | 0.66 | 0.75 | 0.75 | 0.66 | 0.75 | 0.63 | 0.50 | 0.63 | 9 |
| Ciprofloxacin | SVM | 0.96 | 0.89 | 0.96 | 0.95 | 0.88 | 0.96 | 0.96 | 0.89 | 0.96 | 0.95 | 0.89 | 0.96 | 0.94 | 0.84 | 0.94 | 1 |
| Ciprofloxacin | RF | 0.88 | 0.82 | 0.88 | 0.91 | 0.83 | 0.92 | 0.88 | 0.82 | 0.88 | 0.89 | 0.82 | 0.89 | 0.83 | 0.74 | 0.84 | 3 |
| Ciprofloxacin | PLS-DA | 0.87 | 0.75 | 0.90 | 0.88 | 0.75 | 0.91 | 0.87 | 0.75 | 0.90 | 0.87 | 0.75 | 0.90 | 0.82 | 0.64 | 0.86 | 4 |
| Ciprofloxacin | PCA-LDA | 0.76 | 0.69 | 0.79 | 0.79 | 0.70 | 0.80 | 0.76 | 0.69 | 0.79 | 0.77 | 0.69 | 0.79 | 0.66 | 0.55 | 0.70 | 8 |

| | | | | | | | | | | | | | | | | | |
|---|---|---|---|---|---|---|---|---|---|---|---|---|---|---|---|---|---|
| Ciprofloxacin | PCA-GradBoost | 0.84 | 0.86 | 0.85 | 0.87 | 0.86 | 0.87 | 0.84 | 0.86 | 0.85 | 0.85 | 0.86 | 0.86 | 0.78 | 0.80 | 0.79 | 5 |
| Ciprofloxacin | PCA-LogReg | 0.80 | 0.73 | 0.82 | 0.79 | 0.71 | 0.80 | 0.80 | 0.73 | 0.82 | 0.80 | 0.72 | 0.81 | 0.72 | 0.63 | 0.74 | 7 |
| Ciprofloxacin | PCA-SVM | 0.91 | 0.87 | 0.95 | 0.91 | 0.85 | 0.96 | 0.91 | 0.87 | 0.95 | 0.91 | 0.86 | 0.95 | 0.88 | 0.81 | 0.93 | 2 |
| Imipenem | GradBoost | 0.75 | 0.68 | 0.76 | 0.75 | 0.68 | 0.77 | 0.75 | 0.68 | 0.76 | 0.75 | 0.68 | 0.76 | 0.73 | 0.67 | 0.75 | 6 |
| Imipenem | LogReg | 0.62 | 0.60 | 0.61 | 0.62 | 0.60 | 0.61 | 0.62 | 0.60 | 0.61 | 0.62 | 0.60 | 0.61 | 0.59 | 0.59 | 0.58 | 9 |
| Imipenem | SVM | 0.88 | 0.86 | 0.92 | 0.88 | 0.86 | 0.92 | 0.88 | 0.86 | 0.92 | 0.88 | 0.86 | 0.92 | 0.88 | 0.86 | 0.92 | 1 |
| Imipenem | RF | 0.81 | 0.79 | 0.83 | 0.81 | 0.79 | 0.83 | 0.81 | 0.79 | 0.83 | 0.81 | 0.79 | 0.83 | 0.80 | 0.78 | 0.82 | 3 |
| Imipenem | PLS-DA | 0.79 | 0.72 | 0.84 | 0.79 | 0.72 | 0.84 | 0.79 | 0.72 | 0.84 | 0.79 | 0.72 | 0.84 | 0.78 | 0.72 | 0.84 | 4 |
| Imipenem | PCA-LDA | 0.54 | 0.69 | 0.73 | 0.54 | 0.69 | 0.73 | 0.54 | 0.69 | 0.73 | 0.54 | 0.69 | 0.73 | 0.51 | 0.68 | 0.73 | 8 |
| Imipenem | PCA-GradBoost | 0.78 | 0.78 | 0.82 | 0.78 | 0.78 | 0.82 | 0.78 | 0.78 | 0.82 | 0.78 | 0.78 | 0.82 | 0.77 | 0.77 | 0.82 | 5 |
| Imipenem | PCA-LogReg | 0.56 | 0.70 | 0.73 | 0.56 | 0.70 | 0.73 | 0.56 | 0.70 | 0.73 | 0.56 | 0.70 | 0.73 | 0.54 | 0.69 | 0.72 | 7 |
| Imipenem | PCA-SVM | 0.83 | 0.82 | 0.87 | 0.83 | 0.82 | 0.87 | 0.83 | 0.82 | 0.87 | 0.83 | 0.82 | 0.87 | 0.82 | 0.82 | 0.87 | 2 |
| Tobramycin | GradBoost | 0.81 | 0.79 | 0.82 | 0.81 | 0.79 | 0.82 | 0.81 | 0.79 | 0.82 | 0.81 | 0.78 | 0.82 | 0.81 | 0.77 | 0.82 | 6 |
| Tobramycin | LogReg | 0.75 | 0.65 | 0.70 | 0.75 | 0.65 | 0.70 | 0.75 | 0.65 | 0.70 | 0.75 | 0.65 | 0.70 | 0.74 | 0.63 | 0.69 | 7 |
| Tobramycin | SVM | 0.89 | 0.87 | 0.93 | 0.89 | 0.87 | 0.93 | 0.89 | 0.87 | 0.93 | 0.89 | 0.87 | 0.93 | 0.89 | 0.87 | 0.93 | 1 |
| Tobramycin | RF | 0.85 | 0.81 | 0.86 | 0.85 | 0.82 | 0.86 | 0.85 | 0.81 | 0.86 | 0.85 | 0.81 | 0.86 | 0.84 | 0.79 | 0.86 | 3 |
| Tobramycin | PLS-DA | 0.84 | 0.77 | 0.86 | 0.84 | 0.77 | 0.86 | 0.84 | 0.77 | 0.86 | 0.84 | 0.77 | 0.86 | 0.84 | 0.76 | 0.86 | 5 |
| Tobramycin | PCA-LDA | 0.63 | 0.72 | 0.67 | 0.64 | 0.72 | 0.67 | 0.63 | 0.72 | 0.67 | 0.63 | 0.72 | 0.67 | 0.61 | 0.71 | 0.66 | 9 |
| Tobramycin | PCA-GradBoost | 0.84 | 0.82 | 0.84 | 0.84 | 0.82 | 0.84 | 0.84 | 0.82 | 0.84 | 0.84 | 0.82 | 0.84 | 0.83 | 0.82 | 0.83 | 4 |
| Tobramycin | PCA-LogReg | 0.66 | 0.73 | 0.64 | 0.66 | 0.73 | 0.64 | 0.66 | 0.73 | 0.64 | 0.66 | 0.73 | 0.64 | 0.66 | 0.72 | 0.63 | 8 |
| Tobramycin | PCA-SVM | 0.83 | 0.85 | 0.88 | 0.83 | 0.85 | 0.88 | 0.83 | 0.85 | 0.88 | 0.83 | 0.85 | 0.88 | 0.82 | 0.84 | 0.88 | 2 |

### *Supplementary Table 4: Analytical pipelines and hyperparameters for the nine classifiers applied to the single- and multi-excitation spectral datasets*

Supplementary Table 4 **Analytical pipelines and hyperparameters for the nine classifiers applied to the single- and multi-excitation spectral datasets.** A range of classifiers were applied to each of the three spectral datasets to determine the highest performing model for the five bacterial characterisation tasks. To avoid data leakage and enable reliable evaluation of performance estimates, for all classifiers the associated pipeline was fitted to the training set and used to transform the testing set. Additionally, a hyperparameter optimisation was conducted for each classifier to determine the model with the highest macro-averaged F1 score. Acronyms: number of principal components (nPCs), singular value decomposition (SVD), least squares solution (LSQR), eigenvalue decomposition (EGVD), number of latent variables (nLVs).

| Classifier | Pipeline | Hyperparameters |
|---|---|---|
| PCA-LDA | Standard Scalar > PCA > LDA | PCA:<br>nPCs ∈ {5, 10, 15, 20}<br>LDA:<br>solver ∈ {SVD, LSQR, EGVD} |
| PLS-DA | Standard Scalar > PLS-DA | PLS-DA:<br>nLVs ∈ {5, 10, 15, 20},<br>Threshold ∈ {0.25, 0.5, 0.75} |
| SVM | Standard Scalar > SVM | SVM<br>c ∈ {0.01, 0.1, 1},<br>Kernel ∈ {Linear, *RBF*} |
| PCA-SVM | Standard Scalar > PCA > SVM | PCA:<br>nPCs ∈ {5, 10, 15, 20}<br>SVM:<br>c ∈ {0.01, 0.1, 1},<br>Kernel ∈ {Linear, *RBF*} |
| LogReg | Standard Scalar > Select k Best > LogReg | Select k Best<br>k ∈ {10, 50, 100}<br>LogReg:<br>Penalty ∈ {L1, L2, L1 and L2},<br>c ∈ {0.01, 0.1, 1, 10},<br>L1 ratio ∈ {0.25, 0.50, 0.75} |
| PCA-LogReg | Standard Scalar > PCA > LogReg | PCA:<br>nPCs ∈ {5, 10, 15, 20}<br>LogReg:<br>Penalty ∈ {L1, L2, L1 and L2},<br>c ∈ {0.01, 0.1, 1, 10},<br>L1 ratio ∈ {0.25, 0.50, 0.75} |
| GradBoost | Select k Best > GradBoost | Select k Best:<br>k ∈ {10, 50, 100}<br>GradBoost:<br>Learning Rate ∈ {0.05, 0.1},<br>Max Estimator Depth ∈ {2, 3} |

| | | |
|---|---|---|
| PCA-GradBoost | PCA > GradBoost | PCA:<br>nPCs ∈ {5, 10, 15, 20}<br>GradBoost:<br>Learning Rate ∈ {0.05, 0.1},<br>Max Estimator Depth ∈ {2, 3} |
| RF | Select k Best > RF | Select k Best:<br>k ∈ {10, 50, 100, 200, 400, 600, 800, 1000}<br>RF:<br>No. of estimators ∈ {100, 200},<br>Max Tree Depth ∈ {None, 5, 10, 20},<br>Min Samples Split ∈ {2, 5}<br>Min Samples Per Leaf ∈ {1, 2}<br>Max No. Of Features ∈ {square root, $\log_2$} |

## *Supplementary Table 5: Strain and Antibiotic-Sensitivity Identification SVM Test and Validation Scores*

Supplementary Table 5 **Strain and Antibiotic-Sensitivity Identification SVM Test and Validation Scores.** To check for overfitting, the difference between the macro-averaged F1 scores obtained from the validation and test sets for each characterisation task (i.e. the strain, ceftazidime-sensitivity, ciprofloxacin-sensitivity, imipenem-sensitivity, and tobramycin-sensitivity identification problems) was evaluated for the selected model, SVM. Overfitting typically occurs when a model performs well on the validation and training data and poorly on the unseen test data - indicated by a large positive difference between the two metrics. For all models, a zero or negative difference was obtained indicating models were optimised appropriately.

| | Validation Score | | | Test Score | | | Difference | | |
|---|---|---|---|---|---|---|---|---|---|
| | 532 nm | 785 nm | Multi-Excitation | 532 nm | 785 nm | Multi-Excitation | 532 nm | 785 nm | Multi-Excitation |
| Strain | 0.77 | 0.79 | 0.84 | 0.80 | 0.81 | 0.87 | -0.04 | -0.02 | -0.03 |
| Ceftazidime | 0.89 | 0.86 | 0.91 | 0.89 | 0.86 | 0.91 | 0.00 | -0.01 | 0.00 |
| Ciprofloxacin | 0.93 | 0.87 | 0.95 | 0.95 | 0.89 | 0.96 | -0.03 | -0.02 | -0.01 |
| Imipenem | 0.86 | 0.85 | 0.89 | 0.88 | 0.86 | 0.92 | -0.02 | -0.01 | -0.03 |
| Tobramycin | 0.88 | 0.86 | 0.92 | 0.89 | 0.87 | 0.93 | -0.01 | -0.01 | -0.01 |
